# Neoplastic transformation of breast epithelial cells by genotoxic stress

**DOI:** 10.1186/1471-2407-10-343

**Published:** 2010-06-30

**Authors:** Mahendran Botlagunta, Paul T Winnard, Venu Raman

**Affiliations:** 1 Department of Radiology, Johns Hopkins University School of Medicine, Baltimore, Maryland, USA; 2 Department of Oncology, Johns Hopkins University School of Medicine, Baltimore, Maryland, USA

## Abstract

**Background:**

Exposure to genotoxic stresses such as radiation and tobacco smoke can cause increased cancer incidence rate as reflected in an in depth meta-analysis of data for women and breast cancer incidence. Published reports have indicated that exposures to low dose radiation and tobacco smoke are factors that contribute to the development of breast cancer. However, there is a scarcity of information on the combinatorial effects of low dose radiation and tobacco smoke on formation and progression of breast cancer. The combination of these two genotoxic insults can induce significant damage to the genetic material of the cells resulting in neoplastic transformation.

**Methods:**

To study the effects of low dose ionizing radiation and tobacco smoke on breast cells, MCF 10A cells were treated either with radiation (Rad - 0.1 Gray) or cigarette smoke condensate (Csc - 10 microgram/ml of medium) or a combination of Rad + Csc. Following treatments, cells were analyzed for cell cycle distribution patterns and the ability to extrude the Hoechst 33342 dye. In addition, *in vitro *invasion and migration as well as mammosphere formation assays were performed. Finally, differential gene expression profiles were generated from the individual and combination treatment.

**Results:**

Exposure of MCF 10A cells to the combination of radiation plus cigarette smoke condensate generated a neoplastic phenotype. The transformed phenotype promoted increased mammosphere numbers, altered cell cycle phases with a doubling of the population in S phase, and increased invasion and motility. Also, exclusion of Hoechst 33342 dye, a surrogate marker for increased ABC transporters, was observed, which indicates a possible increase in drug resistance. In addition, changes in gene expression include the up regulation of genes encoding proteins involved in metabolic pathways and inflammation.

**Conclusions:**

The results indicate that when normal breast cells are exposed to low dose radiation in combination with cigarette smoke condensate a phenotype is generated that exhibits traits indicative of neoplastic transformation. More importantly, this is the first study to provide a new insight into a possible etiology for breast cancer formation in individuals exposed to low dose radiation and tobacco smoke.

## Background

Women who are exposed to genotoxic stresses such as radiation and tobacco smoke have increased cancer incidence rate as reflected in an in depth meta-analysis of data for cancer incidence [1-5]. In particular, flight attendants exhibit an increased risk of breast and acute myeloid leukemia cancers [1] as they are exposed to long-term doses of low-frequency electromagnetic fields [2-4]. It is well established that doses of low energy radiation can induce double stranded DNA breaks that result in altered gene expression profiles in mammalian cells, which are transmitted to later generations of progeny cells [6]. This lateral transfer of aberrant genomic damage can accelerate the DNA damage rate in subsequent generations, which has been referred to as a radiation induced bystander effect [7,8]. Low dose ionizing radiation has also been shown to alter the intracellular production of reactive oxygen species (ROS), such as, hydrogen peroxide, superoxide anion and hydroxyl radicals [9], which induce mutations and chromosomal aberrations in cells [10]. These types of genetic alterations can promote many pathological conditions including those associated with aging and cancer [11,12]. Such radiation also can dysregulate the expression of stress related proteins and oncoproteins. For example, a number of cellular proteins such as transcription factors (c-Jun, c-fos, IL1, egr-1), cell cycle control (p53, cyclin A and B), and DNA metabolizing proteins (PCNA, β polymerase, PARP) have been shown to be elevated following low dose irradiation [13-17]. Hence, it can be inferred that long term exposure to low dose ionizing radiation can initiate the carcinogenesis process [18].

Besides low dose radiation, there is also a growing body of evidence supporting the hypothesis that exposure to tobacco smoke is a contributing factor in neoplastic transformation of breast cells [5]. Environmental tobacco smoke has been shown to contain high amounts of polycyclic aromatic hydrocarbons (PAHs) many of which have been shown to be potent carcinogens [19-21]. In a rat model, exposure to PAHs rapidly induced palpable mammary tumors [22]. Histological analysis revealed a high incidence of adenocarcinoma indicating the potent carcinogenic property of PAHs. In addition, exposure of human mammary epithelial cells (HMECs) and breast cancer cell lines to an activated PAH: racemic anti-3,4-dihydroxy-1,2-epoxy-1,2,3,4 tetrahydrobenzo phenanthrene (BPDE), found in active and passive cigarette smoke, exhibited altered cell cycle progression, decreased BRCA-1 expression, an increased a spectrum of p53 mutations [23-26] and neoplastic transformation [5]. Additionally, BRCA1 and BRCA2 mutational carriers, who are also smokers, are at an increased risk of getting breast cancer [27]. In contrary, there is evidence to indicate that active smoke does not increase risk of breast cancer in a cohort Japanese women [28].

The combined effect of long-term human exposure to cigarette smoke in combination with ionizing radiation is not known. Thus, the purpose of this research was to determine the combined effects of radiation and cigarette smoke on the ontogeny of breast cancer formation and progression. Towards this goal, we have found that exposure of non-tumorigenic immortalized MCF 10A breast cells to low dose radiation and cigarette smoke condensate promoted a transformed phenotype. These results provide a new insight into a possible etiology for breast cancer formation in populations such as medical staff, aircrews, nuclear test participants and nuclear industry workers that are exposed to low dose radiation and simultaneously may have the likelihood to be exposed to active and second hand cigarette smoke.

## Methods

### Cell culture and exposure to radiation and cigarette smoke condensate

MCF 10A cells (ATCC, Rockville, MD) were irradiated at 0.67 cGy/min to the desired dose (0.1 Gy) using a γ-cell 40 ^137^Cesium irradiator (Atomic Energy of Canada). A 40 mg/ml stock solution of cigarette smoke condensate (Murthy Pharmaceuticals, USA) was prepared in DMSO and diluted with culture medium to a final concentration of 10 mg/ml. Exponentially growing cells were first irradiated and then exposed to Csc (10 μg/ml) for 72 hr [29].

### Flow cytometry and immunoblot analysis

Determination of DNA content by flow cytometry was performed as previously described [30]. Briefly, 2 × 10^6 ^MCF 10A cells were incubated for 72 hr following individual and combined treatments. Fixed cells were incubated with a staining solution containing 0.56% NP-40, 3.7% formaldehyde, and 0.01 mg/ml Hoechst 33258 in phosphate-citrate buffered (pH 7.2) solution. DNA content was analyzed by flow cytometry instrumentation (BD Biosciences, San Jose, CA).

For immunoblot experiments, 20 μg of total cellular protein was subjected to SDS-PAGE. Membranes were probed with the primary antibodies indicated in the figure legend.

### Invasion and wound healing

Matrigel (100 μl; 7-8 mg/ml) in serum-free medium was added to each well of a Transwell Corning Costar plate (Costar, Acton, MA, USA) and dried overnight in a tissue culture hood. The following day, 2.5 × 10^4 ^cells in serum-free medium were pipetted onto the Matrigel and complete medium was added to the bottom trough. Following incubation, the transmembrane filter was stained with crystal violet and the number of cells counted.

For wound healing, a small area was cleared along a diameter of the 10 cm dishes of confluent monolayers of MCF 10A and MCF 10A treated cells with a sterile pipette tip. Cell migration was measured and photographed from the wound/scratch edge every 8 hr.

### Hoechst 33342 dye exclusion assay

Following treatments, cells were incubated in 0.01 mg/ml of Hoechst 33242 dye for 45 minutes and then washed and incubated for a further 45 minutes and photographed using the Nikon 80i fluorescent microscope. Fluorescence intensity per cell (blue channel) was analyzed using image J software (n = 4 in each case).

### Affymetrix analysis

RNA was extracted using the Qiagen mRNA extraction kit. The RNA samples were analyzed with Affymetrix GeneChip Human 133 2.0 Arrays. The quality of the microarray experiments was assessed with *affyPLM *and *Affy*, found at Bioconductor http://www.bioconductor.org. All computation was performed under *R *environment http://www.r-project.org. The *Affy *software was used to estimate the gene expression signals and evaluated using the Robust Multi-array Average [31]. Data normalization was performed using a Bayes method at Bioconductor, which includes log normal modeling. *EBarrays *was used to estimate the posterior probabilities of the differential expression of genes between the control and treated sample [32,33]. This data has been deposited with Gene Expression Omnibus (GEO-accession number GSE21066).

### Generation of mammospheres

Single cell suspensions (total 5000 cells in 2 ml) in Dulbecco's modified Eagle's medium/F-12 containing 5 mg/mL insulin, 0.5 mg/mL hydrocortisone, 2% B27 (Invitrogen Ltd., Paisley, Scotland), and 20 ng/mL epidermal growth factor were seeded into ultra-low attachment plates (Corning, Lowell, MA) and incubated for 7-10 days in presence of 20% O_2 _and 5% CO_2 _at 37°C. Subsequently, the number of mammospheres formed were counted using a microscope.

## Results

### Effects of low dose ionizing radiation and cigarette smoke condensate on MCF 10A cells

**Figure 1:**
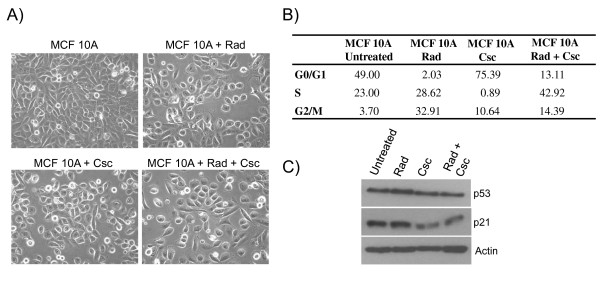
**Phenotypic characterization and cell cycle analyses of MCF 10A cells treated with radiation and cigarette smoke condensate**. **Panel A **- Photomicrographs of MCF 10A-Rad + Csc treated cells (elongated) as compared to the parental MCF 10A cells (cuboidal). **Panel B **- Shows the percentage of cells distributed at the different phases of the cell cycle. **Panel C **- Immunoblot analyses of cell extracts from the control and treated MCF 10A cells. Primary antibodies were against p53 (Santa Cruz) and p21 (Cell Signaling). β-actin (Sigma) was scored for loading control.

The effect of low dose ionizing radiation and cigarette smoke condensate was tested using MCF 10A normal immortalized breast cells. Following treatments, we observed differences between the morphological appearances of the differently treated cultures. Single treatment regimes generated consistent changes in cellular morphologies relative to the untreated controls (Figure 1A). Thus, irradiation or Csc treatment resulted in populations of cells with a rounded or slightly elongated morphology (Figure 1A top right and lower left panels). In contrast, combined treatment (Rad + Csc) of MCF 10A cells generated a phenotype that was somewhat fibroblast-like (Figure 1A lower right panel), which grew with fewer cell to cell contacts and was less cuboidal than the parental cells. Flow cytometry analyses were performed to evaluate whether the latter phenotypic changes could be associated with a change in cell growth rate or cell cycle frequency and hence a change in proliferation rate. As shown in Figure 1B, irradiation (0.1 Gray) increased the percentage of cells found in G2/M from the 3.7% found in the untreated cells to 39.2% while Csc treatment increased the percentage of cells in G0/G1 from 49% found in untreated cells to 75.4%. Thus, irradiation did not cause G1 arrest for MCF 10A cells while Csc treatment caused G1 arrest. However, treatment of MCF 10A cells with a combination of radiation plus Csc resulted in 42.9% of the cells being in S phase, which is roughly double that of the untreated cells and provides an indication that the combined treatment increased the percentage of proliferating cells (Figure 1B). Subsequently, immunoblot analyses were performed to investigate whether the cell cycle changes mediated by genotoxic stress could be correlated to the expression of cell cycle regulatory proteins. As indicated in Figure 1C, p53 protein levels were slightly increased following low dose irradiation, which was matched to an increase in the levels of its downstream target p21. In contrast to Rad, Csc or a combination of radiation plus Csc treatment resulted in a down regulation of p53 and p21 levels (Figure 1C). This indicates that Rad + Csc can alter the levels of regulatory molecules to dysregulate phases of the cell cycle.

### Migration and invasive capabilities of treated MCF 10A cells

**Figure 2:**
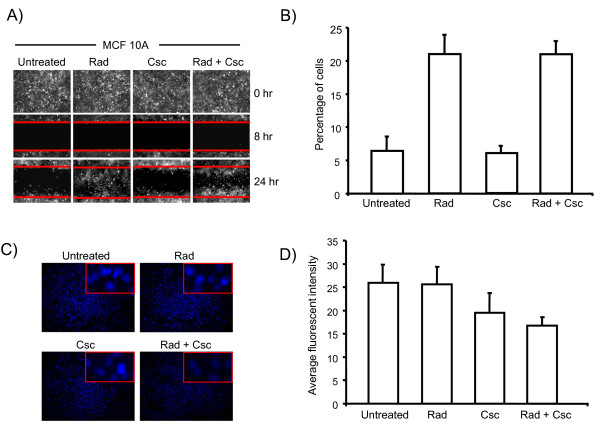
**Invasion and wound healing assays of the treated MCF 10A cells**. **Panel A **- *In vitro *wound healing assay. A filling of the cleared area, as demarcated by the horizontal red lines, was followed microscopically at 20× magnification. **Panel B **- Bar graph quantifying invasion of treated and untreated MCF 10A cells using Boyden chambers (means, n = 2). **Panel C **- Efflux of Hoechst 33342 in MCF 10A treated cells. Inset shows 40× magnification of representative cells. **Panel D **- Bar graph showing quantification of the fluorescence intensity per cell in the blue channel.

Increased cell motility and invasion are characteristics of a neoplastic transformation. To test whether the genotoxic stresses used in our experimental setting could increase the migration of MCF 10A cells, we used a scratch/wound assay. As shown in Figure 2A, MCF 10A cells treated with Rad as well as Rad + Csc were able to migrate into the cleared area within 24 hr of incubation. On the other hand, untreated and Csc treated MCF 10A cells showed little migration into the cleared region during this time period. We next investigated the effect of radiation and Csc on cell invasion in a modified Boyden chamber assay. As depicted in Figure 2B, MCF 10 A cells treated with Csc lack the ability to invade whereas Rad or the combination Rad + Csc cells were extremely invasive (four fold increase) as compared to the untreated and Csc treated MCF 10A cells. Taken together these data indicate that the Rad as well as Rad + Csc treated cells acquired genetic/biochemical alterations that increased the cells ability to migrate and invade, i.e., a possible increase in dissemination or metastatic potential.

### MCF 10A treated cells exhibit increase efflux of Hoechst 33342 dye

A distinct characteristic of a metastatic phenotype is generally increased drug resistant properties. Some of the pathways that promote drug resistance are elevated expression of ATP-binding cassette (ABC) glycoprotein transporters at the cell surface [34]. The elevated expression of these transporters can exclude vital dyes such as Hoechst 33342 analogues to chemotherapeutic drugs, thus promoting multi-drug resistance. As MCF 10A cells treated with Rad + Csc induced a transformed phenotype, we evaluated the ability of these cells to extrude the Hoechst 33342 dye, a surrogate marker for drug uptake. As seen in Figure 2C &2D, cells treated with both Rad + Csc retained less dye as compared to the untreated MCF 10A cells. This indicates that the combination treatment in MCF 10A cells can induce a drug resistant phenotype.

### Changes in gene expression profile and mammosphere formation following genotoxic stress

**Figure 3:**
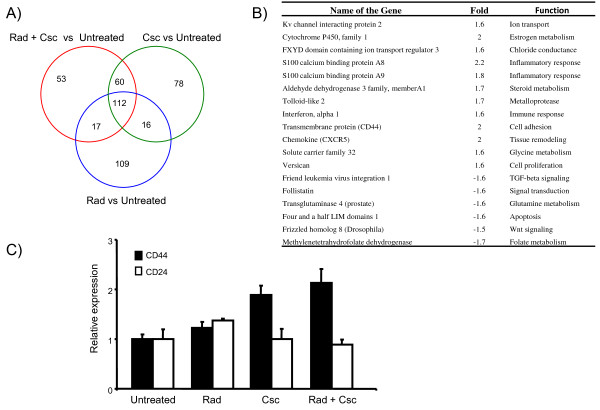
**Differential gene expression pattern in MCF 10A cells following genotoxic stress**. **Panel A **- Venn diagram depicting the total number of genes that intersect following exposure of MCF 10 A cells to Rad, Csc and Rad + Csc. **Panel B **- Select set of up and down regulated genes, at 72 hr following treatment, that intersect in Rad + Csc conditions. **Panel C **- CD44 and CD24 levels as determined by the real time PCR from RNA samples extracted from cell lines following Rad, Csc and Rad + Csc treatment.

**Figure 4:**
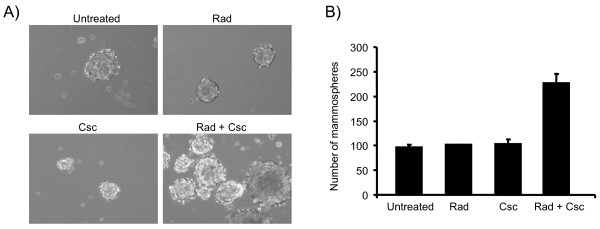
**Mammosphere formation of MCF 10A cells treated with radiation and cigarette smoke condensate**. **Panel A **- Photomicrographs of the mammospheres following treatment with Rad, Csc and Rad + Csc. **Panel B **- Shows the total number of mammospheres following exposure to Rad, Csc and Rad + Csc. The counts represented are averages from two independent experiments.

In an effort to determine which genes might be contributing to the increased cell invasion and migration capabilities of Rad or Rad + Csc treated MCF 10A cells, we performed expression analyses using the Affymetrix platform. To identify differentially expressed gene sets following treatment, we compared the individual (Rad or Csc) treatment data sets with the combined treatment (Rad + Csc) data set using a Venn diagram (Figure 3A). The Venn diagram indicates that 53 genes differentially expressed in the combination treated cells compared to untreated cells. Among these, 18 genes (12 up regulated and 6 down regulated) have been reported as participating in cellular pathways of cancer (Figure 3B). Included in this group were genes involved in tissue remodeling, metabolism and cell adhesion molecules. One cell adhesion molecule identified was CD44, which has been directly correlated to human breast cancer grade [35]. Moreover, a high CD44 low CD24 expression profile in breast cancer cell lines has been associated with a putative breast cancer initiating cell phenotype [36]. In order to validate the microarray data, we estimated the CD44 levels in treated versus untreated cell lines. The expression level of CD44 was up regulated 2 fold in the combined treatment as compared to the untreated and Rad treated (Figure 3C). Recently, cells exhibiting a high CD44 low CD24 expression profile have been shown to form mammospheres in culture conditions, which were resistant to chemotherapeutic regimes [37]. Therefore, we investigated the effect of genotoxic stress on MCF 10A cells to form mammospheres *in vitro*. Figure 4A shows representative mammosphere images under different genotoxic stress. The combined treated cells formed the most number of mammospheres as compared to individual treatments (Figure 4B). Thus, the Affymetrix data indicates that the combined treatment can initiate gene expression patterns that are associated with known cancers. That along with enhanced mammosphere formation strengthens our suggestion that further experiments will indicate that the altered gene profiles are consistent representations of known cancer cell phenotypes.

## Discussion

Long-term exposure to low dose radiation at levels that can damage normal cell functions and propagate dsyregulated molecular programs can generate diseased tissue. For example, aircrews, medical staff and workers in the nuclear energy industry are often exposed to low dose radiation as well as cigarette smoke and the combined effect of these two environmental stresses likely identifies these as a few of the populations that have an increased risk of developing cancer. As low dose radiation and cigarette smoke exposure may very well alter the breast tissue biochemistry, physiology, and morphology, it is essential to determine if the combined effects of such exposures induces genetic/biochemical alterations that can trigger breast tumor formation. Such considerations led us to study the action of the combined environmental genotoxic stresses of low dose radiation and cigarette smoke condensate on immortalized non-tumorigenic breast epithelial cells, MCF 10A. The finding that exposing cells to both Rad + Csc generated cells that exhibited a fibroblastic phenotype, which is different from the normal cuboidal shaped MCF 10A cells, is a clear indication that fundamental genetic alterations occurred during treatment. The low dose irradiation used in this study induced cell cycle arrest (G2/M), which is in agreement with this well known effect occurring in cells exposed to radiation [38]. Although, we observed only marginal increases in p53 and p21, these changes nevertheless support the known function of these proteins in this check point process [39]. However, Csc and combined treated cells reversed or abrogated the check point response to ionizing radiation and caused a shift in cell cycle to G0/G1 and S phase respectively with concomitant decreases in p53 and p21. The alterations in the cell cycle distribution pattern following combined treatment indicates that a combination of genotoxic exposures is more detrimental with respect to the generation of a proliferating phenotype then either single exposure under the conditions used here.

Tumorigenesis is a complex process and it involves intricate biological mechanisms such as invasion and motility that are essential attributes of metastasis [40]. We observed that Rad or Rad + Csc doubled the percentage of cells that invaded the Matrigel. We have further demonstrated that exposure of MCF 10A cells to low dose radiation followed by Csc altered the transcription profiles of a number of genes that fell into two broad categories: (1) those with common responses across individual treatments and (2) those with differential responses associated with combined treatment. Our study is the first genome-wide analysis of transcript profiles associated with concomitant radiation and cigarette smoke condensate exposures. The major changes associated with the differential response can be categorized as those cellular properties associated with tissue remodeling, metabolism, and altered cell membrane protein levels, while a more ambiguous outcome was observed for genes involved in inflammation and signaling events. Our findings of increased transcript levels in a large number of metabolic genes appear to indicate that metabolism is an important component of stress response mechanisms after exposure to radiation or Csc. An increase in Cytochrome P450 observed in Rad + Csc treated cells may metabolically activate the carcinogens present in cigarette smoke within the breast environment to promote carcinogenesis. Furthermore, cell membrane proteins have been previously reported to be elevated after low dose radiation exposures [41]. The significant changes in the expression level of CD44 and mammosphere number induced by Rad + Csc supports the generation of radio-resistant phenotype as compared to individual treated fraction [37].

Although a direct correlation of results obtained on cultured human cells to the human situation requires additional research, we envisage that the results of the present study will help initiate further studies on the affects of long term human exposure to low dose radiation and tobacco smoke in the pathogenesis of breast cancer.

## Conclusions

In conclusion, we have demonstrated that short term exposure of normal breast cells to low doses of ionizing radiation and the potent carcinogens in tobacco smoke condensate can induce genetic and phenotypic changes that reflect those of known cancer cell types. Given the limitations of these in vitro studies and the complexities of translating these findings to real life responses to such environmental mutagens and carcinogens is a strong indication that further investigation will be required to conclusively demonstrate the risk of getting breast cancer following exposure to low dose radiation and cigarette smoke. Still this is the first study to demonstrate the effects of low dose ionizing radiation in combination with exposures to tobacco smoke carcinogens that can result in a possible initiation of cellular processes that give rise to breast cancer cells.

## Abbreviations

Rad: radiation; Csc: cigarette smoke condensate; ABC: ATP-binding cassette; ROS: reactive oxygen species.

## Competing interests

The authors declare that they have no competing interests.

## Authors' contributions

MB collected and analyzed the data and drafted the manuscript. PW helped in the data analysis and manuscript writing. VR conceived the study and participated in data analysis, interpretation and manuscript writing. All authors have read and approved the final manuscript.

## Pre-publication history

The pre-publication history for this paper can be accessed here:

http://www.biomedcentral.com/1471-2407/10/343/prepub
